# US-Guided Percutaneous Radiofrequency versus Microwave Ablation for Benign Thyroid Nodules: A Prospective Multicenter Study

**DOI:** 10.1038/s41598-017-09930-7

**Published:** 2017-08-25

**Authors:** Zhigang Cheng, Ying Che, Songyuan Yu, Shurong Wang, Dengke Teng, Huixiong Xu, Jianwei Li, Desheng Sun, Zhiyu Han, Ping Liang

**Affiliations:** 1 Department of Interventional Ultrasound, Chinese PLA General Hospital, Beijing, 100853 China; 2 Department of Ultrasound, the First Affiliated Hospital of Dalian Medical University, Dalian, 116011 Liaoning Province China; 3 Department of Ultrasound, Tianyou Hospital Affiliated to Wuhan University of Science & Technology, Wuhan, 430046 Hubei Province China; 4 Department of Ultrasound, Yantai Affiliated Hospital of Binzhou Medical University, Yantai, 264100 Shandong Province China; 5 Department of Interventional Ultrasound, Chinese PLA No. 208 Hospital, Changchun, 130062 Jilin Province China; 6 Department of Medical Ultrasound, Shanghai Tenth People’s Hospital, Ultrasound Research and Education Institute, Tongji University School of Medicine, Shanghai, 200072 China; 7 Department of Ultrasound, Fujian Provincial Hospital, Fuzhou, 350001 Fujian Province China; 8 Department of Ultrasound, Peking University Shenzhen Hospital, Shenzhen, 518036 Guangdong Province China

## Abstract

Compared with microwave ablation (MWA), percutaneous radiofrequency ablation (RFA) and laser ablation (LA) have been recommended as minimally invasive treatments for patients with symptomatic benign thyroid nodules (BTNs) because of the large number of clinical applications. This prospective multicenter study sought to evaluate the clinical outcomes of RFA and MWA for BTNs. In eight participating institutions, the total number of 1252 patients treated by RFA and MWA were 649 ones with 687 BTNs and 603 ones with 664 BTNs, respectively. The clinical outcomes including the nodular maximal diameter reduction ratio (MDRR), the nodular volume reduction ratio (VRR), and the incidence of complications were compared to evaluate the efficacy and safety of the two techniques. The results for the nodular MDRR and VRR in the RFA group were significantly better than those in the MWA group at 6 months and later follow-up, and the major complication rates of 4.78% and 6.63% in RFA and MWA groups showed no statistically significant differences. In conclusion, both RFA and MWA are safe and effective techniques for selected patients with symptomatic BTNs. The achieved MDRR and VRR in the RFA group were greater than those in the MWA group at 6 months and later follow-up.

## Introduction

The thyroid nodule is one of the most common lesions in clinical practice^1^ and has been increasingly detected in approximately 50% of the general population by ultrasound (US) examination^2^ in the past two decades due to the widespread use of radiological imaging. Benign thyroid nodules (BTNs) proven cytologically by fine-needle aspiration biopsy (FNAB) account for 85 to 95% of all thyroid nodules^3^. It is unnecessary to treat most BTNs because of their lack of symptoms and slow growth^4^. Therapy is mainly focused on patients with progressive nodular growth or cosmetic problems and symptoms, which are related to nodular volume^5^. Therefore, it is essential for the treatment of BTNs to maximize the nodular volume reduction ratio (VRR) and minimize complications because of the anatomical features of the thyroid and its adjacent important structures.

After over a decade of clinical application, percutaneous radiofrequency ablation (RFA) and laser ablation (LA) under US guidance have become minimally invasive treatments for patients with BTNs, especially for nonsurgical candidates, surgical high-risk individuals or those refusing surgery^5, 6^. Low complication rates and high nodular VRRs for RFA and LA have been confirmed in the treatment of BTNs^7–9^, and the preservation of thyroid function^10, 11^ after ablation has been more encouraging in clinical applications. Comparing the clinical outcomes of RFA and LA for BTNs, similar safeties have been observed because of the similar incidences of major and minor complications. However, the nodular VRR for RFA has been shown to be slightly superior to that for LA^12^ and RFA has been shown to have the highest probability (98.7%) of being the more effective treatment^13^, although no randomized controlled trial with large-scale cases and long-term follow-up has been published.

A large-scale clinical study with a short-term follow-up period, followed by a pilot study using a modified microwave antenna^14^, of microwave ablation (MWA) for 477 BTNs (mean index nodule volume, 2.1 ml) in 222 patients was published. The mean VRR was 65% at 6-month and the major complication rate was 3.6% (8/222). MWA seems to be a safe and effective technique for the treatment of BTNs^15^. A recent interesting study^16^ retrospectively compared the clinical outcomes of RFA and MWA and concluded that both of them were effective and safe methods in treating BTNs. The purpose of the present prospective multicenter study was to further evaluate the clinical outcomes of the two techniques.

## Design of the study

This prospective multicenter non-randomized trial compared the clinical outcomes after a single ablation procedure of RFA vs. MWA in a series of patients with BTNs.

## Materials and Methods

### Patients

This prospective multicenter study was approved by the Institutional Ethics Committees (IECs) of Chinese PLA General Hospital (Beijing, China, registration number [2013] 010-02), the First Affiliated Hospital of Dalian Medical University (Dalian, China), Tianyou Hospital Affiliated to Wuhan University of Science & Technology (Wuhan, China), Yantai Affiliated Hospital of Binzhou Medical University (Yantai, China), Chinese PLA No. 208 Hospital (Changchun, China), Shanghai Tenth People’s Hospital (Shanghai, China), Fujian Provincial Hospital (Fuzhou, China) and Peking University Shenzhen Hospital (Shenzhen, China). All methods were performed in accordance with the approved guidelines and regulations and the study was authorized by the Chinese Clinical Trial Registry (Number: ChiCTR-ONRC-13003087, 20/02/2013, http://www.chictr.org.cn/enIndex.aspx). The manuscript was drafted according to the STROBE statement (http://www.strobe-statement.org/). A total of eight institutions participated in this multicenter study, where there was at least one interventional US physician with five years of experience, a special operating room and over five hundred interventional US procedures annually. Both RFA and MWA were applied in four centers each, and the admitted patients were assigned to the RFA group and MWA group, respectively. Written informed consent was obtained from each patient before enrollment.

Inclusion criteria included BTNs that were proven cytopathologically at least twice or histopathologically once and presented with (1) maximal diameter no less than 2 cm, progressive growth and solid component more than 20%; (2) symptomatic problems such as neck pain or discomfort, foreign body sensation, or compressive symptoms; (3) cosmetic problems; (4) clinical thyrotoxicosis and hyperthyroidism caused by autonomously functioning thyroid nodules (AFTNs); (5) no prior therapy. Patients with severe conditions, malignant or follicular thyroid neoplasms, previous surgery or medicine for the thyroid, and vocal cord palsy in the side contralateral to the target nodules were excluded.

### US and ablation equipment

Four types of real-time US equipment were used in the participating centers, which included the Sequoia 512 system (Acuson, Mountain View, CA, USA), the Logiq E9 (GE Healthcare, Milwaukee, WI, USA), the Aplio™ 500 ultrasound machine (Toshiba Medical Systems Co. Ltd, Otawara, Japan), and the Hi Vision Preirus ultrasonography system (Hitachi Medical (Guangzhou) Co., Ltd., Guangzhou, China). High-resolution linear probes (6–12 MHz) were employed to monitor and guide the biopsies, pre-ablation evaluation, ablation procedures and follow-up.

The RFA system of the VIVA RF generator (VIVA RF generator, STARmed, Goyang, Korea) was applied in the study. An 18-gauge, monopolar, modified, internal-cooled RFA electrode (VIVA, STARmed, Goyang, Korea) with a 1-cm active tip and a 7-cm shaft length was applied, which was specifically developed for ablation of thyroid nodules^17^.

The MWA system used in the study the KY-2000 2450 MHz microwave system (KY-2000, Kangyou Medical, Nanjing, China). A 16-gauge, Teflon-coated, internal-cooled microwave antenna with a 3-mm active tip and a 10-cm shaft length was applied, which was specifically modified for the ablation of thyroid nodules^14, 15^.

### Pre-ablation evaluations

#### Symptomatic and cosmetic problems

The symptomatic scores in patients with BTNs could be self-measured by the patients with a 10-cm visual analogue scale (scores ranged from 0 to 10). The cosmetic scores were evaluated by an experienced physician and ranged from 1 to 4: (1) no palpable mass; (2) palpable mass but no cosmetic problems; (3) cosmetic problem on swallowing only or detected by an experienced physician; and (4) a readily detected cosmetic problem^14^.

#### Pathological confirmation

To determine the diagnosis of a BTN prior to ablation, at least two separate cytopathological examinations by FNAB or one histopathological examination by core needle biopsy (CNB) were necessary^6^.

#### Imaging examinations

US examination played a key role in characterizing the target nodule and distinguishing the relationship between the nodule and the important surrounding anatomic structures. The maximal diameter (a) and two orthogonal diameters (b and c), echogenic features, internal vascularity and the proportion of the solid component of each nodule on US were assessed by experienced operators. The nodular volume (V) was estimated by the ellipsoid formulation V = πabc/6. According to the US presentation of the internal nodule on color Doppler flow imaging (CDFI), the nodular vascular scores were classified into four grades: (1) no color signal in the nodule; (2) color signals in <25% of the nodule; (3) color signals in 25–50% of the nodule; and (4) color signals in >50% of the nodule^18^. Examinations of the bilateral vocal cords in all patients were performed with a laryngo-fiberscope before ablation. For AFTNs, a technetium ^99^mTc pertechnetate thyroid scintigraphy was recommended for careful evaluation.

#### Laboratory examinations and anticoagulation agents

Laboratory examinations including serum infectious indexes, a complete blood count, blood coagulation, serum calcium, phosphorous, calcitonin and glucose were routinely performed before the ablation. The thyroid function indexes such as thyroid stimulating hormone (TSH), triiodothyronine (T3) and free thyroxine (fT4) were needed to test before the ablation. Anticoagulation and antiplatelet agents were temporarily omitted 5–7 days prior to the ablation and could be retaken after to the procedure^19^.

### Procedure

To objectively compare the applications of MWA and RFA, only one ablation session was performed to a target nodule and its clinical outcomes were recorded, analyzed and followed. If two or more target nodules in one patient were located in two lobes of the thyroid, two separate sessions were performed.

Except for the differences in ablation devices and electrodes between RFA and MWA, similar procedures were performed in each center. To ensure the safety of the procedure, fasting at least 8 hours and the establishment of a venous catheter in a forearm vein of the patient prior to the procedure were requested.

The patient was in a supine position with a small pillow under the neck to expose the cervical region sufficiently. A multi-parameter monitor continuously monitored the patient’s vital signs such as the blood pressure, the partial pressure of oxygen, the pulse rate and the electrocardiogram during the procedure. All procedures were completed under a sterile operation and local anesthesia with 1% lidocaine. With the appropriate pressure of the US probe, the target nodule and its adjacent structures were clearly identified on real-time US and then the pre-procedure planning including the best approach to the target nodule was confirmed.

When the nodule was located in the upper or lower pole of the thyroid or adjacent to vital structures such as the vagus nerve, trachea or esophagus, hydrodissection^20^ could be a helpful technique to prevent unexpected nerve injury. A 21-gauge needle was introduced percutaneously between the thyroid capsule near the target nodule and its surrounding structures, and then 0.9% saline (for MWA group) or 5% dextrose solution (for RFA group) was injected to separate the thyroid from the surrounding structures.

The cystic component in the nodule was aspirated by an 18-gauge percutaneous puncture needle during the procedure. After the antenna or electrode was advanced into the target nodule under US guidance, the moving shot technique^6^ was employed to complete the ablation procedure. Power outputs of 20–50 W and 25–60 W were usually applied in MWA and RFA, respectively. For the portion of rich vascularity in the nodule, the fixed electrode technique or high output power was used to block the blood flow. During the procedure, the patient’s voice should be heard at intervals by several questions and answers between the operator and the patient. If a voice change was detected, the procedure was stopped immediately. Hemorrhage was detected in a timely manner by real-time US. The ablation was paused, and then, serious supervision or emergent management was required if a massive hemorrhage occurred.

After ablation, a small ice bag was used to cover the front of the patient’s neck for 6 hours and at least 30 minutes of observation was necessary. The vital signs of patients were normal and no early complications were detected concerning the standards of the patient’s leave.

### Post-ablation evaluations and follow-up

US was the first-line imaging administered to assess changes in the ablated nodules, including their sizes, volumes, vascularity, echogenicity, undertreated portions and regrowth, by experienced sonographers in each center. The US presentation of the ablated nodules, the symptomatic and cosmetic scores and laboratory tests such as T3, fT4, TSH of the patients were evaluated and recorded at the 3rd, 6th, and 12th months of follow-up in the first year after ablation and 6–12 months thereafter.

The volume changes in BTNs before and after ablation on US presentation were assessed by the maximal diameter reduction ratio (MDRR) and VRR formulated as follows: (1) maximal diameter reduction ratio = (initial maximal diameter - final maximal diameter) × 100/initial maximal diameter (%); (2) VRR = (initial volume - final volume) × 100/initial volume (%).

The clinical efficacies of the RFA and MWA groups were evaluated by comparing MDRR, VRR, vascular, symptomatic and cosmetic scores, therapeutic success rates (defined as the number of ablated nodules with VRR > 50% at the last follow-up, accounting for the percentage of the total number of ablated nodules in each group)^20^, nodular complete disappearance rates (defined as the number of ablated nodules undetected by US at the last follow-up, accounting for the percentage of the total number of ablated nodules in each group) and nodular regrowth rates (defined as the number of the ablated nodules with an increase in nodular volume more than 50% of the previous follow-up volume, accounting for the percentage of the total number of ablated nodules in each group)^18^.

According to the reporting standards of the Society of Interventional Radiology^21, 22^, to compare the safety of the RFA and MWA groups, complications, including hemorrhage, voice change, skin burn, hypothyroidism, hyperthyroidism and nodule rupture, and side effects, such as pain, coughing and mild fever, in the peri-ablation and follow-up periods were recorded.

### Statistical analysis

Data analysis was performed with SPSS statistical software, version 22.0 (SPSS Inc. Chicago, IL, USA), and GraphPad Prism software version 5.0 for windows (GraphPad Software, San Diego, California, USA) was applied to draw the graphs. Values for quantitative variables were expressed as the mean ± SD (range). In the MWA or RFA group, one-way ANOVA and the Duncan’s test were used to compare the nodular maximal diameter and volume and the vascular, symptomatic and cosmetic scores between pre-procedure and at each follow-up.

Between the RFA and MWA groups, an independent-samples t-test was performed to compare the age, the mean ablation energy, time and power during the procedure, the nodular MDRR and VRR at each follow-up, and the vascular, symptomatic and cosmetic scores pre-procedure and after each follow-up. The χ^2^ test was applied for categorical variables, including gender distribution, the number of patients with multiple-nodules, the number of patients achieving therapeutic success, the number of regrown nodules, the number of nodules with complete disappearance, minor or major complications and side effects between the two groups.

All the tests were two sided. P values less than 0.05 were considered significant differences.

### Data availability statement

All data generated or analysed during this study are included in this published article and its supplementary information files.

## Results

### Patients

From January 2013 to December 2015, 1289 patients with BTNs were admitted in eight institutions according to the inclusion criteria and underwent RFA or MWA procedures. Thirty-seven patients (2.9%) without any follow-up after the ablation were excluded. A total of 1,252 patients with 1,351 nodules but no AFTNs were enrolled in the present study, and the number of nodules treated with RFA and MWA were 687 in 649 cases and 664 in 603 cases, respectively. All patients’ thyroid hormones including TSH, T3 and fT4 were at normal ranges before ablation. The clinical features of the BTNs in the two groups before treatment are listed in Table 1. The number of the enrolled patients and BTNs and operators’ experience in the eight participating institutions are listed in Table 2.

**Table 1:** The enrolled patients and clinical characteristics of BTNs pre-procedure for the RFA and MWA group.

Characteristics	RFA group	MWA group	P value
No. of patients/BTNs	649/687	603/664	—
Gender (M/F) (n)	140/509	135/468	0.733
Age (years)	47.9 ± 13.6(12–87)	47.1 ± 12.9(12–79)	0.295
Pts. with uni-/multi-nodules (n)	607/42	552/51	0.196
Maximal diameter (cm)	2.91 ± 0.71(2–6.1)	2.92 ± 0.93(2–6.3)	0.819
Volume (ml)	7.22 ± 6.76(0.71–52.56)	7.72 ± 9.16(0.38–70.16)	0.270
Vascular score	2.22 ± 0.84(1–4)	2.31 ± 0.83(1–4)	0.081
Symptomatic score	0.44 ± 1.03(0–4)	0.47 ± 1.10(0–4)	0.602
Cosmetic score	2.27 ± 1.02(1–4)	2.33 ± 0.90(1–4)	0.231
Follow-up (months)	13.5 ± 6.3(3–24)	13.9 ± 6.0(3–24)	0.179

Values are presented as the mean ± SD (range). BTNs: benign thyroid nodules, RFA: radiofrequency ablation, MWA: microwave ablation, M/F: male/female, Pts.: patients, cm: centimeter, ml: milliliter.

**Table 2:** Summary of demographical data and operators’ experience in the eight participating institutions.

Participating Institutions	No. of Pts.	No. of nodules	Ablation	Operators	Operators’ experience (years)	
					Thyroid US examination	Thyroid ablation
The First Affiliated Hospital of Dalian Medical University	535	572	RFA	Y. C.	20	5
Tianyou Hospital Affiliated to Wuhan University of Science & Technology	207	218	MWA	S. Y.	20	5
Chinese PLA General Hospital	179	197	MWA	P. L.	30	10
				Z. C.	20	5
				Z. H.	20	5
Yantai Affiliated Hospital of Binzhou Medical University	166	188	MWA	S. W.	20	5
Chinese PLA No. 208 Hospital	51	61	MWA	D. T.	20	5
Shanghai Tenth People’s Hospital	49	49	RFA	H. X.	20	5
Fujian Provincial Hospital	44	45	RFA	J. L.	20	5
Peking University Shenzhen Hospital	21	21	RFA	D. S.	20	5
Total	1252	1351	—	—	—	—

Pts: Patients; US: Ultrasound; RFA: Radiofrequency ablation; MWA: Microwave ablation.

### Procedure

Because of the target BTNs located in bilateral lobes of thyroid, two separate sessions were performed for 3.2% (21/649) patients in RFA group and 5.0% (30/603) ones in MWA group, respectively. Therefore, for all the participating centers, the total number of treatment sessions was 1303 and hydrodissection was performed in 457 sessions (35.1%). One session was performed for 628 and 573 patients and two sessions were performed for 21 and 30 patients in RFA and MWA groups, respectively. The mean ablation energy, power output and ablation time for BTNs of the RFA group vs. the MWA group were 8,751.7 ± 9,481.9J (ranged from 1,750 to 83,475 J) vs. 9,136.6 ± 6,707.7J (ranged from 800 to 51,300 J), 50.8 ± 9.8 W (ranged from 25 to 60 W) vs. 32.3 ± 7.9 W (ranged from 20 to 50 W), and 193.4 ± 237.5 s (ranged from 35 to 1,855 s) vs. 289.5 ± 208.9 s (ranged from 20 to 1,470 s), respectively. There was no statistical significance for ablation energy (p = 0.391) in RFA and MWA groups, but the ablation time (p = 0.000) and power output (p = 0.000) showed significant differences (Supplementary Table S1).

Mild pain at the ablation area or pain radiating to the head, ear, shoulder or teeth was commonly observed during the procedure for either RFA or MWA, especially when the ablated lesion was close to the skin, but quickly released when the energy emission was decreased or lessened or the active tip was removed. No patients were required to cease the procedure because of intolerance in the study.

### Efficacy evaluation at follow-up

**Figure 1:**
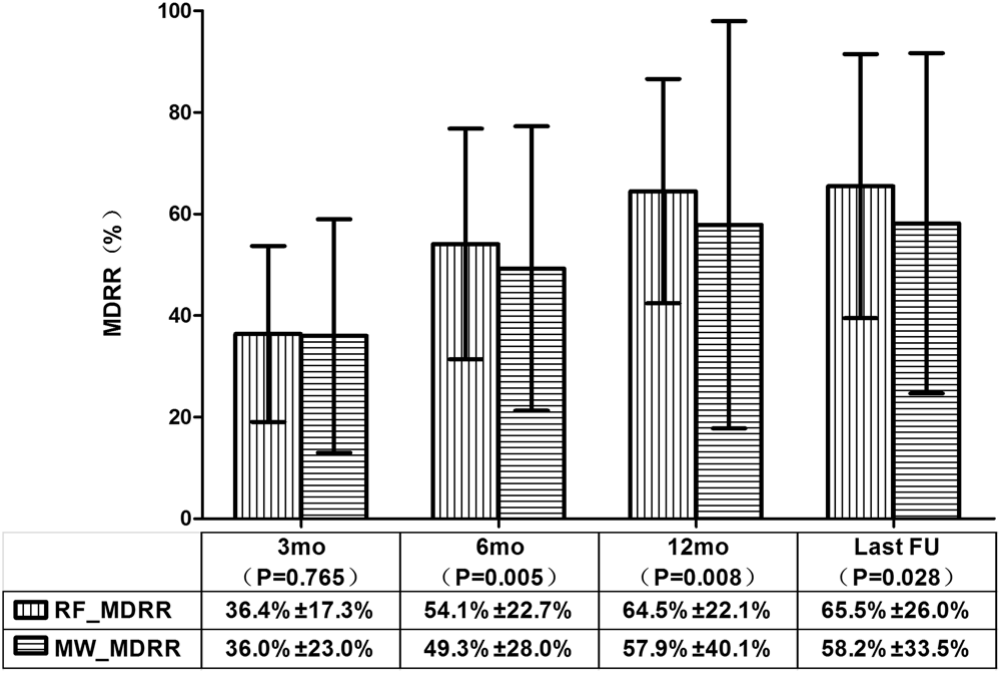
The comparison of the ablated nodular MDRRs between the RFA and MWA groups at each follow-up period. At 3-month follow-up, the mean MDRR between the two groups showed no significant differences (p = 0.765), but after that, statistical significance was observed, and a higher MDRR was found in the RFA group relative to the MWA group. The mean ± standard deviation is illustrated. (MDRR: maximal diameter reduction ratio; RFA: radiofrequency ablation; MWA: microwave ablation; RF_MDRR: the mean nodular MDRR in the RFA group; MW_MDRR: the mean nodular MDRR in the MWA group; mo: months; FU: follow-up).

In the RFA group, the mean maximal diameters of the ablated nodules at the 3rd, 6th, and 12th month and the last follow-up were 1.87 ± 0.77 cm, 1.38 ± 0.86 cm, 1.12 ± 0.87 cm, and 1.04 ± 0.78 cm, respectively, all of which were significantly less than that before ablation; the P values were less than 0.001. In the MWA group, the mean maximal diameters of the ablated nodules at the 3rd, 6th, and 12th month and the last follow-up were 1.96 ± 0.92 cm, 1.51 ± 0.92 cm, 1.25 ± 1.19 cm, and 1.34 ± 1.17 cm, respectively, all of which were significantly less than that before ablation; the P values were less than 0.001. The mean MDRRs of the RFA vs. the MWA groups at the 3rd, 6th, and 12th month and the last follow-up showed in Fig. 1.

**Figure 2:**
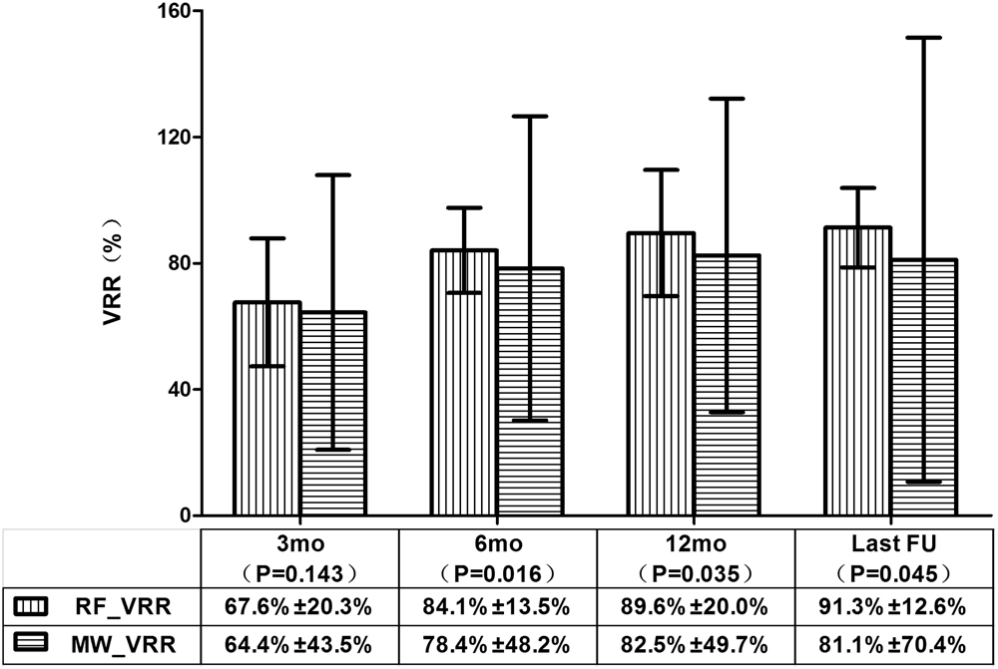
The comparison of the ablated nodular VRRs between the RFA and MWA groups at each follow-up period. At 3-month follow-up, the mean VRR between the two groups showed no significant differences (p = 0.143), but after that, statistical significance was observed and a higher VRR was shown in the RFA group than in the MWA group. The mean ± standard deviation is illustrated. (VRR: volume reduction ratio; RFA: radiofrequency ablation; MWA: microwave ablation; RF_VRR: the mean nodular VRR in the RFA group; MW_VRR: the mean nodular VRR in the MWA group; mo: months; FU: follow-up).

In the RFA group, the mean volumes of the ablated nodules at the 3rd, 6th, and 12th month and the last follow-up were 2.42 ± 3.04 ml, 1.29 ± 2.03 ml, 0.87 ± 1.88 ml, and 0.64 ± 1.23 ml, respectively, all of which were significantly less than that before ablation; the P values were less than 0.001. In the MWA group, the mean volumes of the ablated nodules at the 3rd, 6th, and 12th month and the last follow-up were 2.48 ± 4.06 ml, 1.34 ± 2.80 ml, 0.98 ± 2.39 ml, and 1.25 ± 3.53 ml, respectively, all of which were significantly less than that before ablation; the P values were less than 0.001. The mean VRRs of the RFA group vs. the MWA group at the 3rd, 6th, and 12th month and the last follow-up were 67.6 ± 20.3% vs. 64.4 ± 43.5% (p = 0.143), 84.1 ± 13.5% vs. 78.4 ± 48.2% (p = 0.016), 89.6 ± 20.0% vs. 82.5 ± 49.7% (p = 0.035) and 91.3 ± 12.6% vs. 81.1 ± 70.4% (p = 0.045), respectively (Fig. 2). The mean nodular VRRs at the 3rd month of follow-up showed no significant difference between the two groups, but at the 6th month and later follow-up, statistical significance was found, and the RFA group had a higher VRR than that in the MWA group.

**Figure 3:**
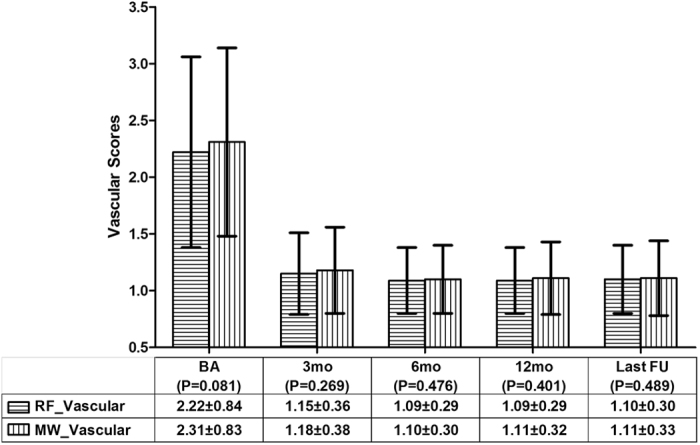
The comparison of the nodular vascular scores between the RFA and MWA groups. There were no significant differences in the vascular scores before the ablation and at the 3rd, 6th, and 12th month and the last follow-up The mean ± standard deviation is illustrated. (RFA: radiofrequency ablation; MWA: microwave ablation; RF_Vascular: the mean vascular score in the RFA group; MW_Vascular: the mean vascular score in the MWA group; BA: before the ablation; mo: months; FU: follow-up).

**Figure 4:**
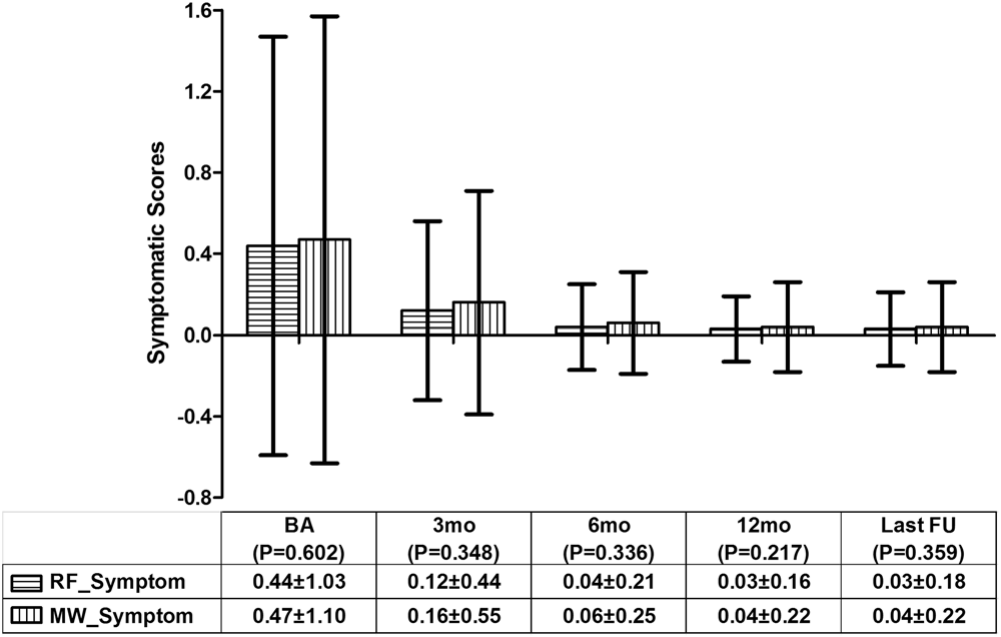
The comparison of the nodular symptomatic scores between the RFA and MWA groups. There were no significant differences in the symptomatic scores before the ablation and at the 3rd, 6th, and 12th month and the last follow-up. The mean ± standard deviation is illustrated. (RFA: radiofrequency ablation; MWA: microwave ablation; RF_Symptom: the mean symptomatic scores in the RFA group; MW_Symptom: the mean symptomatic scores in the MWA group; BA: before the ablation; mo: months; FU: follow-up).

**Figure 5:**
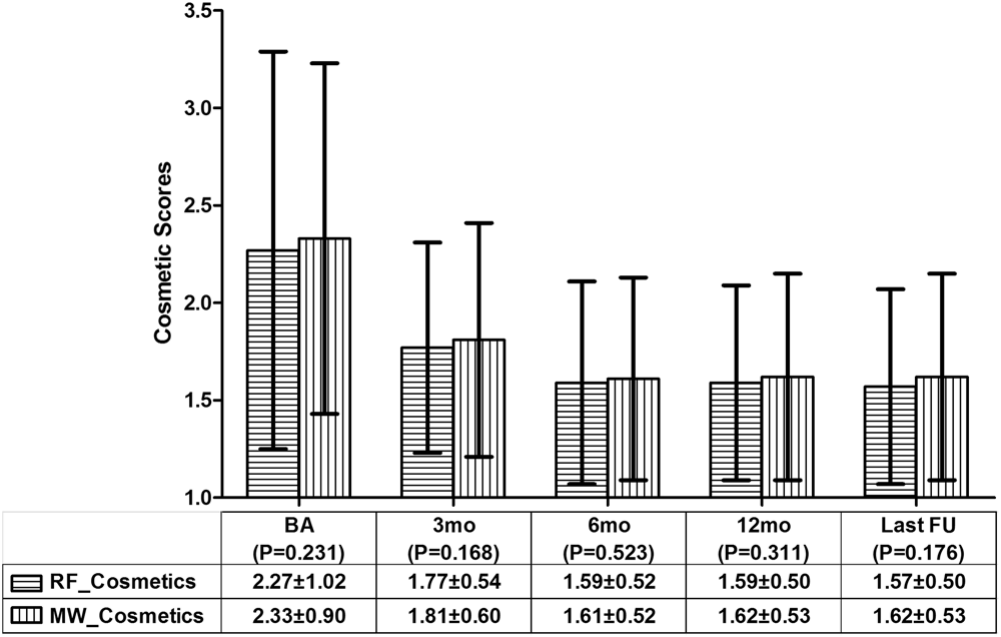
The comparison of the nodular cosmetic scores between the RFA and MWA groups. There were no significant differences in the cosmetic scores before the ablation and at the 3rd, 6th, and 12th month and the last follow-up. The mean ± standard deviation is illustrated. (RFA: radiofrequency ablation; MWA: microwave ablation; RF_Cosmetics: the mean cosmetic scores in the RFA group; MW_Cosmetics: the mean cosmetic scores in the MWA group; BA: before the ablation; mo: months; FU: follow-up).

The mean vascular, symptomatic and cosmetic scores before ablation vs. the last follow-up in the RFA group all significantly decreased; the P values were less than 0.001. The mean vascular, symptomatic and cosmetic scores before ablation vs. the last follow-up in the MWA group were all significantly decreased; the P values were less than 0.001. Between the RFA and MWA groups, there were no significant differences in the mean vascular (Fig. 3), symptomatic (Fig. 4) and cosmetic (Fig. 5) scores at the same observation time.

At the last follow-up, the therapeutic success rates in the RFA and MWA groups were 80.2% (551/687) and 76.4% (507/664), and were presented no significant differences (p = 0.086) between the two groups. The nodular complete disappearance rates of the ablated nodules in the RFA and MWA groups were 23.1% (159/687) and 19.9% (132/664), and showed no statistical significances (p = 0.145) between the two groups. All nodular regrowth was observed in the undertreated peripheral portion of the nodules. The nodular regrowth rates in the RFA and MWA groups were 7.0% (48/687) and 7.7% (51/664), and had no significant differences (p = 0.625) between the two groups.

### Safety evaluation at follow-up

All the complications and side effects encountered in the peri-ablation and follow-up periods in the two groups are listed in Table 3. The major and minor complication and side effect rates for the RFA group vs. the MWA group all showed no statistically significant differences, with 4.78% vs. 6.63% (p = 0.156), 2.00% vs. 2.49% (p = 0.562) and 4.93% vs. 4.64% (p = 0.812), respectively.

**Table 3:** The complications and the side effects in the RFA and MWA groups in the peri-ablation and follow-up periods.

Complications or side effects	RFA group No. (%)	MWA group No. (%)	P value
Major complications	31 (4.78)	40 (6.63)	p = 0.156
Voice change	29 (4.47)	35 (5.80)	
Nodule rupture with/without infection	2 (0.31)	4 (0.66)	
Sympathetic nerve injury	0 (0)	1 (0.17)	
Minor complications	13 (2.00)	15 (2.49)	p = 0.562
Hemorrhage/hematoma	13 (2.00)	12 (1.99)	
Vomiting	0	1 (0.17)	
Skin burn	0	1 (0.17)	
Hyperthyroidism	0	1 (0.17)	
Side effects	32 (4.93)	28 (4.64)	p = 0.812
Pain with oral analgesics	20 (3.08)	17 (2.82)	
Coughing	0	1 (0.17)	
Mild fever (37–38.1)	12 (1.85)	10 (1.66)	

RFA: radiofrequency ablation, MWA: microwave ablation.

Major complications were observed in 71 patients in the study. The most common major complications were voice changes, with an incidence of 5.1% (64/1252). The voice change in 38 (3.0%) of 64 cases recovered within 3 days after the procedure and was relieved in 1 to 3 months in the remaining 26 patients (2.1%) by medications including corticosteroids and physiotherapy. Six patients (0.5%) experienced nodule rupture, which occurred 1 to 3 months following the procedure. Surgery was performed in one patient (0.1%) out of the 6 patients because of local infection, and the remaining 5 patients (0.4%) recovered 1 to 2 months after oral antibiotics and/or local drainage. Sympathetic nerve injury^23^ occurred in one patient (0.1%) presenting mild ipsilateral ptosis after the ablation of a right thyroid nodule and the patient recovered within 1 month with oral mecobalamin treatment.

Twenty-eight (2.2%) out of 1252 patients exhibited minor complications. Local hemorrhage or hematoma formation were the most common ones during thyroid nodule ablation and were observed in 25 (2.0%) out of 1252 patients, who recovered within one week without sequelae. Vomiting and skin burn after the procedure occurred in one patient (0.1%). The TSH level of a 21-year-old male patient (0.1%) was lower than normal range, and T3 and fT4 were slightly higher than the normal range in the thyroid function test, but no clinical symptoms of hyperthyroidism were found at the 6th and 9th month of follow-up. No medication was prescribed after consultation with the endocrinologist and the levels of TSH, T3 and fT4 returned to normal ranges at the 12th month of follow-up. No hypothyroidism occurred in any of the patients during the follow-up.

Side effects, including pain with oral analgesics, coughing and mild fever with a temperature less than 38.1 °C, could recover without treatment within 1 to 3 days after ablation.

## Discussion

The present large population multicenter study enrolled 1,252 patients with 1,351 nodules and achieved mean follow-up period of 13 month with 2.9% follow-up loss. In order to avoid the biases come from two or more sessions and obtain objectively comparative results for the clinical evaluation of the efficacy and safety of percutaneous RFA and MWA treatments for BTNs under US guidance, only one ablation session was performed on a target nodule in both groups according to the trail protocols of this multicenter study.

For MDRR and VRR, no significant differences were found after 3 months between the two groups, while the RFA group achieved better results than the MWA group at the 6th and 12th month and the last follow-up. This could be explained by the basic principles of RFA and MWA. Because of the high central temperature in tissue, usually more than 150 °C^24^, which was commonly produced by MWA^25^, carbonization in the ablated lesion could easily occur, even at a relatively low power output, such as 30–50 W^15^. However, the central temperature produced by RFA was not more than 110 °C^26^, and the presentation of carbonization in an RFA lesion is very rare. It is essential that the increases in MDRR and VRR related to the necrosis of tissue in the ablated nodules disappear gradually. The carbonized tissue in the ablated nodules was difficult to dissolve, and the MDRR and VRR of the ablated nodules in the RFA group exhibited greater increases than those in the MWA group. According to previous studies, the mean VRRs of RFA, LA and MWA were 89.9 ± 10.2%^18^, 72 ± 11%^27^ and 65 ± 65%^15^, respectively. A recent retrospective study^16^ concluded that the VRRs achieved by RFA and MWA for the treatment of BTNs at 1, 3, 6 and 12 months follow-up were no statistical significance. To compare with the results of our study, except the influence of the number of the enrolled patients, a low microwave power output of 20–35 W might contribute to minimize the carbonization in the ablated nodules and increase the VRR of MWA group at follow-up.

Decreasing the power output during the MWA procedure might be an effective method to increase the MDRR and VRR when treating BTNs. In the present study, the mean power output for MWA was significantly less than that for RFA; however, it was still not enough to minimize carbonization. Thus, 20–30 W of microwave power output would be recommended in further applications. One of the possible causes of the longer procedure time in MWA was that microwaves need to be carried in waveguides, such as coaxial cables, which are typically more cumbersome than the fine wires used to feed energy to RF electrodes^24^ and are inconvenient when performing the freehand moving shot technique. Furthermore, whether BTNs with a rich blood flow would be beneficial for the high central temperature during the MWA procedure should be further evaluated by a comparative clinical study.

In our study, all nodular regrowth was observed in the undertreated peripheral portion of the nodules which was adjacent to the important structures such as recurrent laryngeal nerve. The nodular regrowth could usually be detected by experienced operators via different US echogenicity at ablated portion. Color Doppler US or contrast enhanced US were helpful to distinguish the nodular regrowth portion in the ablated nodules according to the presentations of blood flow signal or contrast agent perfusion there. Additionally, the nodular regrowth were commonly slow^18^ and regular observation was recommended in our study. Finally, repeat FNAB played a key role when the regrowth nodule was considered the possibility of malignancy^18^.

To evaluate the safety, the incidences of complications and side effects, especially major complications, were at low levels in both groups in the present study. Between the RFA and MWA groups, the major and minor complications and the side effects presented no statistical significance. For major complications, voice changes after ablation were the most common complaints. One of the important causes of to result in voice changes is nerve injury, especially injury of the laryngeal recurrent nerve during the ablation procedure. However, voice changes might be caused by hemorrhage^7^ or temperature increase instead of lethality to the nerves due to thermal conduction during the ablation of large nodules, and patients would recover within several days post-ablation without any treatment. In this study, patients, who suffered voice changes after ablation and completely recovered within 1 to 3 days, accounted for 28.1% (18/64) in the RFA group and 31.3% (20/64) in the MWA group of all patients with voice change. Voice changes caused by the above reasons were all recorded and statistically analyzed as one of major complications in the protocols of the prospective study, and the incidence seemed higher than the rates of 1.4%, 3.6% and 0.5% in previous studies on RFA^7^, MWA^15^ and LA^27^, respectively. Several effective points could be helpful to minimize the complication of voice change after thyroid ablation as follows. The first was that a careful US examination was required for the target nodule and the adjacent cervical structures, especially to pay more attention along the directions of the cervical nerves. The second was that several short-term pauses during the thermal ablation could be adopted to decrease the thermal conduction, especially in the ablation procedure of large BTNs; and then the patient could be asked to answer a short question at the interval to confirm whether or not the voice change occurred. The third was that the energy would be fired only after the active tip of the ablation needle was clearly monitored in the target nodule on the US imaging. The last but not the least, although the present prospective study used hydrodissection technique, incidence of voice change was slightly higher than those of other studies. In thyroid ablation, injected fluid disappears rapidly along the neck space; therefore continuous injection of fluid is necessary during ablation. The minor complication rate in the present study was close to those that occurred in the previously published studies including RFA^7^ and LA^27^.

Sympathetic nerve injury resulting in Horner syndrome, which clinically presents as a combination of ptosis, miosis, and anhidrosis of the face at the ipsilateral side of the injury nerve, is a very rare complication of RFA or MWA in treating BTNs^23^. The possible reason of this complication is the middle cervical sympathetic ganglion usually located at the lower pole of the thyroid was damaged during thermal ablation^23^. To minimize the complication, firstly, careful US examination of the adjacent structures around the target nodule plays an important role to distinguish the middle cervical sympathetic ganglion^28^. Secondly, hydrodissection is an effective method to separate the target nodule and the surrounding important structures, especially for the target nodule located at the lower pole of the thyroid. Thirdly, US-guided cautious tracing of the ablation needle and the active tip never advanced beyond the margin of the target nodule are mandatory during RFA or MWA procedure^6^.

There were several limitations in our study. The first was that even though a prospective multicenter study was designed, a randomized controlled trial was not employed because it was difficult to randomize the enrolled patients in the eight hospitals. The second was that no comparison between RFA or MWA and surgery for the treatment of BTNs was reported and a further prospectively randomized controlled trial with long-term follow-up comparing ablation techniques and surgery would offer more convincing evidence for the clinical application of thermal ablation in treating BTNs. The third originated from the false negative rate of CNB for the diagnoses of thyroid nodules^29^. Only one time CNB was not enough to confirm the diagnosis of BTN, although no malignant nodules were found during follow-up in this study. The fourth was that this study included not only solid nodules but also predominantly cystic thyroid nodules. Current evidences recommend ethanol ablation as a first-line treatment for predominantly cystic nodules rather than thermal ablations^5^. Including predominantly cystic thyroid nodules can increase VRR. The last but not the least was that this study enrolled small size thyroid nodules (mean volume less than 10 ml) and achieved only short-term follow-up (mean follow-up period, 13 month); therefore further study is necessary for medium (10–20 ml) or large (more than 20 ml) size nodules^30^ and longer follow-up.

## Conclusion

Both RFA and MWA under percutaneous US guidance are safe and effective techniques for selected patients with BTNs due to their low incidence of complications and side effects, significant shrinkage of the nodular volumes and the significant improvement of patients’ symptoms and cosmetic concerns related to the nodules. Larger MDRR and VRR can be achieved in the RFA group than those in the MWA group at 6 months and later follow-up.

## Electronic supplementary material

Supplementary file
