# Supplementary material for: US-Guided Percutaneous Radiofrequency versus Microwave Ablation for Benign Thyroid Nodules: A Prospective Multicenter Study
Source: Sci Rep. 2017 Aug 25;7:9554. doi: 10.1038/s41598-017-09930-7 (PMC5573330; doi:10.1038/s41598-017-09930-7)
Supplement: Supplementary file 1 — Supplementary file [file 41598_2017_9930_MOESM1_ESM.pdf]

US-Guided Percutaneous Radiofrequency versus Microwave Ablation for Benign Thyroid Nodules: A Prospective Multicenter Study

Zhigang Cheng[1], Ying Che[2], Songyuan Yu[3], Shurong Wang[4], Dengke Teng[5], Huixiong Xu[6], Jianwei Li[7], Desheng Sun[8], Zhiyu Han[1], and Ping Liang[1*]

[1] Department of Interventional Ultrasound, Chinese PLA General Hospital, Beijing, 100853, China

[2] Department of Ultrasound, the First Affiliated Hospital of Dalian Medical University, Dalian, 116011, Liaoning Province, China

[3] Department of Ultrasound, Tianyou Hospital Affiliated to Wuhan University of Science & Technology, Wuhan, 430046, Hubei Province, China

[4] Department of Ultrasound, Yantai Affiliated Hospital of Binzhou Medical University, Yantai, 264100, Shandong Province, China

[5] Department of Interventional Ultrasound, Chinese PLA No. 208 Hospital, Changchun, 130062, Jilin Province, China

[6] Department of Medical Ultrasound, Shanghai Tenth People's Hospital, Ultrasound Research and Education Institute, Tongji University School of Medicine, Shanghai, 200072, China

[7] Department of Ultrasound, Fujian Provincial Hospital, Fuzhou, 350001, Fujian Province, China

[8] Department of Ultrasound, Peking University Shenzhen Hospital, Shenzhen, 518036, Guangdong Province, China

*Correspondence to: Ping Liang, MD, Professor of Medicine, Chief, Department of Interventional Ultrasound, Chinese PLA General Hospital, No. 28, Fuxing Road, Haidian District, Beijing, 100853, China, liangping301@126.com. Telephone: +86-10-66939530.

Supplementary Table S1 The ablation parameters for BTNs in the procedures for the RFA and MWA group

Ablation parameters	RFA group	MWA group	P value
Sessions (one/two)	628/21	573/30	0.152
Energy (J)	8,751.7±9,481.9 (1,750-83,475)	9,136.6±6,707.7 (800-51,300)	0.391
Power output (W)	50.8±9.8(25-60)	32.3±7.9(20-50)	0.000
Time (s)	193.4±237.5(35-1,855)	289.5±208.9(20-1,470)	0.000

Values are presented as the mean ± SD (range). BTNs: benign thyroid nodules, RFA: radiofrequency ablation, MWA: microwave ablation, J: joule, W: Watt, s: second.
